# Physical activity levels associated with insomnia and depressive symptoms in middle-aged and elderly patients with chronic schizophrenia

**DOI:** 10.3389/fpsyt.2022.1045398

**Published:** 2023-01-06

**Authors:** Zhiwei Liu, Yulong Zhang, Liang Sun, Juan Wang, Lei Xia, Yating Yang, Feng Sun, Wenzheng Li, Xianhu Yao, Rongchun Yang, Huanzhong Liu

**Affiliations:** ^1^Department of Psychiatry, The Third People’s Hospital of Fuyang, Fuyang, China; ^2^Department of Psychiatry, Chaohu Hospital of Anhui Medical University, Hefei, China; ^3^Anhui Psychiatric Center, Anhui Medical University, Hefei, China; ^4^Department of Psychiatry, Chengdu Fourth People’s Hospital, Chengdu, China; ^5^Department of Psychiatry, Hefei Fourth People’s Hospital, Hefei, China; ^6^Department of Psychiatry, Ma’anshan Fourth People’s Hospital, Ma’anshan, China

**Keywords:** physical activity, insomnia, depressive, middle-aged, elderly, schizophrenia

## Abstract

**Background:**

Previous evidence suggested that physical activity had beneficial effects on psychopathological symptoms, insomnia, or depressive symptoms in people with schizophrenia. This study investigated the association between physical activity levels and insomnia and depressive symptoms in middle-aged and elderly hospitalized patients with chronic schizophrenia (CS).

**Methods:**

179 participants were enrolled. We used the 30-item Positive and Negative Syndrome Scale (PANSS_–30_) to assess the psychopathological symptoms. We used the Insomnia Severity Index scale (ISI) and 17-item Hamilton Depression Scale (HAMD-17) to evaluate insomnia and depressive symptoms. Daily physical activity time less than 30 min, within 30–60 min, and more than 60 min were defined as physical inactivity, moderate physical activity, and vigorous physical activity, respectively. The Chi-square test, analysis of variance (ANOVA), and Mann–Whitney *U*-test were applied for categorical, continuous, and non-normal distribution variables, respectively. The Pearson or Spearman’s correlation analyses were utilized to examine the association between physical activity levels, ISI total scores, HAMD total scores, and socio-demographic and clinical variables. Finally, socio-demographic variables with a *P*-value < 0.05 in the comparison between insomnia/depressive group and non-insomnia/depressive group were considered for inclusion in binary logistic regression analysis to determine the relationship between physical activity levels and insomnia or depressive symptoms.

**Results:**

The ISI total scores (*r* = –0.247, *P* = 0.001) and HAMD total scores (*r* = –0.312, *P* < 0.001) were negatively correlated with physical activity levels. Logistic regression analysis revealed that older age, higher depressive factor scores, and lower physical activity level were influential factors of insomnia symptoms in CS patients (*P* < 0.05). In addition, vigorous physical activity (compared with physical inactivity) and higher negative and depressive factor scores were independently associated with depressive symptoms in CS patients (*P* < 0.05).

**Conclusion:**

Physical activity levels were influential factors in comorbid insomnia and depressive symptoms in CS patients. Given the benefits of physical activity, it should be strengthened as a routine adjunct to clinical treatment or psychiatric care so as to improve the physical and mental health of patients with psychiatric symptoms.

## Introduction

World Health Organization (WHO) statistics reported that approximately 15% of older adults aged 60 years and older suffer from mental disorders, such as depression, anxiety, and dementia, making it a category of public health challenges that seriously affects the elderly population (1). The results of the Global Burden of Disease Study (GBD) showed that mental disorders accounted for a significantly increasing proportion of global disability-adjusted life years (DALYs) and 14.6% of global disability life lost years (YLDs), making it one of the top 10 global burdens of disease (2). The latest, the most authoritative the national epidemiological survey of mental disorders in China indicated that the lifetime prevalence of mental illness in adults was 16.57% (3), with alone accounting for approximately 17% of the global burden of mental disorders (4), creating a heavy long-term burden at the individual, societal, and national levels.

Schizophrenia is a group of severe mental disorders of unknown etiology. Patients suffer from serious abnormalities in cognition, thinking, and behavior (5), with a lifetime prevalence of approximately 1% worldwide (6). Previous literature stated that the population with mental disorders suffered poorer sleep quality than the general population, and about 20–40% of patients with chronic schizophrenia (CS) were comorbid insomnia symptoms (7–9). Furthermore, research suggested that worse sleep quality was strongly related to more severe psychotic symptoms (10) and that insomnia symptoms could even exacerbate psychiatric symptoms and lead to increased somatic comorbidity. In contrast, healthy sleep hygiene significantly improved the severity of psychopathological symptoms (11). Strong evidence suggested that regular physical activity enhanced mental health (12) and could predict a better global and social quality of life in schizophrenic patients (13). Furthermore, higher physical activity levels were strongly related to fewer insomnia symptoms, better cognitive functioning, social functioning, and life satisfaction compared to lower levels of physical activity (14).

Comorbid depressive symptoms were more frequent in patients with schizophrenia, with an estimated prevalence of 18.8–80% (15–17). One study noted that depressive symptoms were negatively associated with the severity of psychotic symptoms (16). Compared to patients with mildly depressed schizophrenia, patients with major depressive symptoms had more serious psychiatric symptoms and poorer quality of life (15). Furthermore, a cross-sectional survey showed that more severe depressive symptoms in patients with schizophrenia were strongly related to less daily physical activity (18). Strong evidence suggests that regular physical activity may reduce depressive symptoms and improve social functioning in schizophrenic patients significantly (19, 20).

Physical activity, or physical exercise, sports, etc., is considered to be an important factor related to mental health. The latest meta-analysis shows that participation in sports can significantly reduce the body fat content and improve the physical function and mental health of elderly people over 60 years old (21). Another meta-analysis suggests that aerobic exercise may have a significant effect on mood and anxiety symptoms (22). In addition, clinical study indicates that a combination of high sedentary behavior and low moderate-to-vigorous intensity physical activity (MVPA) is strongly associated with higher levels of depression and anxiety symptoms compared with populations with less sedentary behavior and with sufficient MVPA (23). According to a national survey, higher PA frequency was associated with lower levels of depression, anxiety in Chinese physicians (24). A systematic review of 31 studies found that reducing sedentary behavior and increasing MVPA were significantly associated with better mental health and quality of life (25). Meanwhile, physical activity has also proven to be a promising adjunctive intervention for mood disorders.

Although growing literature evidence reported the correlation between physical activity levels, insomnia symptoms, depressive symptoms and psychopathology, studies on middle-aged and elderly Chinese hospitalized schizophrenic patients are still lacking. This research investigated the association between physical activity levels, insomnia and depressive symptoms in middle-aged and elderly hospitalized patients with schizophrenia. Further, we analyzed the influencing factors of comorbidities of insomnia or depressive symptoms in schizophrenia.

## Materials and methods

### Subjects

The subjects were psychiatric inpatients from May to December 2018 from three tertiary hospitals (Chaohu Hospital of Anhui Medical University, Hefei Fourth People’s Hospital, and Ma’anshan Fourth People’s Hospital) in Anhui Province, China. This cross-sectional study was a secondary analysis of the physiological and psychological conditions of inpatients with CS. Please refer to our previous manuscripts for sample size calculation (26). Inclusion criteria: (1) age ≥ 45 years; (2) it met the diagnostic criteria of schizophrenia according to the 5th edition of the Diagnostic and Statistical Manual of Mental Disorders (DSM-V); (3) disease duration ≥ 5 years; and (4) able to complete clinical scale assessment. Exclusion criteria: (1) the presence of other serious mental retardation or neurological diseases; (2) unable to complete the assessment of clinical symptoms scales; (3) women who are pregnant or breastfeeding; (4) complicated with serious physical diseases (such as cardiovascular diseases, digestive system diseases, respiratory diseases, etc.).

The enrolled patients and their guardians agreed to participate in the project after knowing the study process and the related advantages and disadvantages and signed the paper informed consent. This research was approved by the Ethics Committee of Chaohu Hospital of Anhui Medical University (No. 201805-kyxm-03) and obtained registration number (No. ChiCTR1800017044) from the China Clinical Trials Registry.

### Socio-demographic and clinical data

Demographic variables (age, gender, education, etc.) and clinical information of each patient were collected by questionnaire and the electronic medical record. Antipsychotic dose in chlorpromazine equivalents was calculated using the defined daily dose method (27).

### Blood indicators

Ten milliliters of fasting venous blood was collected on the second morning after enrollment. The fasting blood glucose (FBG), total cholesterol (TC), triglyceride (TG), high-density lipoprotein (HDL-C), low-density lipoprotein (LDL-C), and other blood lipid indexes were detected by a specialized laboratory technician.

### Physical activity levels

According to the dietary guidelines for Chinese residents, it is recommended that middle-aged and older people exercise outdoors one to two times a day for 30–60 min each time (28). In addition, WHO suggested that adults complete 150–300 min of moderate physical activity per week (29). In this study, the daily physical activities included walking, rhythmic exercises, etc., and the activity time was comprehensively judged by patients’ self-reported and medical care records. All subjects were measured by three simple questions and answered the questions “yes” or “no”: (1) Did you participate in any form of physical activity during hospitalization, such as rhythmic exercises, jogging, walking, etc. (2) The amount of physical activity you did each day: less than 30 min, 30–60 min, or more than 60 min. (3) Did you have any physical discomfort when you participate in physical activity? Less than 30 min per day was considered physical inactivity, 30–60 min per day was considered moderate physical activity, and more than 60 min per day was considered vigorous physical activity.

### Assessment of psychiatric symptoms

30-item Positive and Negative Syndrome Scale (PANSS_–30_) was utilized to assess the psychiatric symptoms of each inpatient. The five-factor model consists of positive, negative, cognitive, depressive, and excited factor scores were used for the statistical scoring, respectively (30).

Insomnia Severity Index scale (ISI) was used to assess insomnia symptoms with good reliability and validity (31, 32). The ISI total scores range from 0 to 28. We defined ISI total scores ≥ 8 as comorbid insomnia symptoms (31).

17-Item Hamilton Depression Scale (HAMD_–17_) was used to assess depressive symptoms. Higher scores of HAMD indicate the different degrees of depression. We defined HAMD total scores ≥ 8 as comorbid depressive symptoms (33). The three clinical assessment scales’ intraclass correlation coefficient (ICC) exceeded 0.8.

### Statistical analysis

The SPSS 23.0 software package (*IBM, Chicago, IL, USA*) was used for data analysis. First, Chi-square test, analysis of variance (ANOVA), and Mann–Whitney *U*-test were applied for categorical, continuous, and non-normal distribution variables, respectively. Second, Pearson or Spearman’s correlation analyses were utilized to examine the association between physical activity levels, ISI total scores, HAMD total scores, and socio-demographic and clinical variables. Finally, socio-demographic variables with a *P*-value < 0.05 in the comparison between insomnia/depressive group and non-insomnia/depressive group were considered for inclusion in binary logistic regression analysis to determine the relationship between physical activity levels and insomnia or depressive symptoms. The significance level was set as α = 0.05 (2-tailed).

## Results

### Comparisons between groups with and without insomnia or depressive symptoms

179 inpatients with CS were included, and the prevalence of insomnia or depressive symptoms was 22.9% (41/179) and 71.5% (128/179). Compared to patients without insomnia symptoms, the comorbid insomnia symptoms patients had older age, lower physical activity level, higher proportion of antipsychotic polypharmacy, higher FBG levels, and higher psychopathology symptoms such as positive/depressive factor scores, PANSS, ISI, and HAMD total scores. Compared to patients without depressive symptoms, the patients with depressive symptoms had lower physical activity levels, fewer hospitalizations, higher positive, negative, cognitive, and depressive factor scores, PANSS, ISI and HAMD total scores (see Table 1).

**TABLE 1:** Comparison of clinical and biological indicators between insomnia group and non-insomnia group, depressive group, and non-depressive group.

Variables	Insomnia group (*n* = 41)	Non-insomnia group (*n* = 138)	X^2^/F	*P*	Depressive group (*n* = 128)	Non-depressive group (*n* = 51)	X^2^/F	*P*
Age (years, $\bar{x}\pm s$)	55.71 ± 7.49	52.94 ± 5.97	-2.449	**0**.**015**^a^	52 (49.25, 57)	52.45 ± 5.92	-1.385	0.166^b^
**Gender**								
Male (%)	21 (51.2)	78 (56.5)	0.360	0.549^c^	69 (53.9)	30 (58.8)	0.357	0.550^c^
Female (%)	20 (48.8)	60 (43.5)			59 (46.1)	21 (41.2)		
Education (years)	7.68 ± 3.73	8 (5, 11)	-0.454	0.650^b^	8 (5, 11)	8 (8, 11)	-1.154	0.249^b^
Onset age (years)	29.12 ± 8.67	29.32 ± 8.62	0.128	0.898^a^	29.41 ± 8.56	28.92 ± 8.78	-0.345	0.731^a^
Illness duration (years)	26.39 ± 11.64	23.75 ± 9.78	-1.449	0.149^a^	24.67 ± 10.31	23.57 ± 10.20	-0.648	0.518^a^
Number of admissions	5 (3, 8.5)	5 (3, 9)	-0.017	0.986^b^	5 (3, 8)	8.25 ± 5.82	-2.274	**0**.**023**^**b**^
BMI	24.03 ± 3.48	23.96 ± 3.90	-0.107	0.915^a^	24.05 ± 3.60	23.77 ± 4.27	-0.442	0.659^a^
**Antipsychotic category (%)**								
Monotherapy	9 (22.0)	67 (48.6)	9.154	**0**.**002**^c^	50 (39.1)	26 (51.0)	2.120	0.145^c^
Polypharmacy	32 (78.0)	71 (51.4)			78 (60.9)	25 (49.0)		
**Hematological index**								
FBG (mmol/L)	5.30 ± 1.09	4.80 (4.40, 5.40)	-2.008	**0**.**045**^b^	4.90 (4.50, 5.40)	5.20 ± 1.24	-0.005	0.996^b^
TC (mmol/L)	5.08 ± 2.03	4.69 (4.03, 5.50)	-0.757	0.449^b^	4.74 (4.09, 5.49)	4.86 ± 1.65	-0.413	0.680^b^
TG (mmol/L)	2.30 ± 1.75	1.82 (1.35, 2.59)	-0.326	0.744^b^	1.82 (1.36, 2.52)	1.96 (1.28, 2.93)	-0.272	0.786^b^
HDL-C (mmol/L)	1.06 ± 0.31	0.98 (0.87, 1.19)	-0.888	0.375^b^	1.04 ± 0.27	1.06 ± 0.26	0.422	0.674^a^
LDL-C (mmol/L)	2.54 ± 0.64	2.45 ± 0.65	-0.823	0.412^a^	2.48 ± 0.62	2.45 ± 0.73	-0.267	0.790^a^
**Physical activity levels**								
Physical inactivity	18 (43.9)	38 (27.6)	9.756	**0**.**008**^c^	48 (37.5)	8 (15.7)	12.846	**0**.**002**^c^
Moderate physical activity	20 (48.8)	58 (42.0)			56 (43.8)	22 (43.1)		
Vigorous physical activity	3 (7.3)	42 (30.4)			24 (18.7)	21 (41.2)		
**PANSS**								
Positive factor scores	11.8 ± 4.94	9 (6.75, 14)	-2.019	**0**.**044**^b^	10.5 (7, 15)	9.47 ± 5.40	-2.509	**0**.**012**^**b**^
Negative factor scores	19.66 ± 7.35	17.64 ± 6.61	-1.668	0.097^a^	19.33 ± 6.35	15.04 ± 7.05	-3.951	**<0**.**001**^a^
Cognitive factor scores	9.46 ± 3.23	9.12 ± 2.84	-0.666	0.506^a^	9.57 ± 2.77	8.25 ± 3.14	-2.761	**0**.**006**^a^
Depressive factor scores	7.90 ± 2.91	6 (4, 8)	-2.732	**0**.**006**^b^	7.48 ± 2.84	5.16 ± 1.90	-5.365	**<0**.**001**^a^
Excited factors scores	8.10 ± 3.27	6 (5, 9)	-1.538	0.124^b^	7 (5, 9)	6 (4, 10)	-1.500	0.134^b^
PANSS total scores	84.95 ± 24.45	76.73 ± 22.78	-1.995	**0**.**048**^a^	82.97 ± 21.57	67.69 ± 24.30	-4.125	**<0**.**001**^a^
ISI total scores	11.61 ± 3.29	1.82 ± 1.54	-26.596	**<0**.**001**^a^	3 (1, 8)	1 (0, 2)	-6.012	**<0**.**001**^b^
HAMD total scores	14.49 ± 4.31	8.91 ± 4.45	-7.100	**<0**.**001**^a^	12 (9, 15)	5 (3, 7)	-10.458	**<0**.**001**^b^
Chlorpromazine equivalent (mg/d)	468.23 ± 221.92	360 (227.5, 600)	-1.682	0.093^b^	428.02 ± 223.05	300 (240, 540)	-0.848	0.397^b^
Hypnotics prescription (yes,%)	9 (22.0)	30 (21.7)	0.001	0.977^**c**^	29 (22.7)	10 (19.6)	0.199	0.656^c^
Antidepressants prescription (yes,%)	2 (4.9)	10 (7.2)	0.283	0.594^**c**^	10 (7.8)	2 (3.9)	0.883	0.347^c^

^a^Independent samples *t*-test.

^b^Mann-Whitney *U*-test.

^c^Pearson chi-square. Bold value means *P* < 0.05.

### Correlation analysis between ISI total scores, HAMD total scores, and demographic and clinical variables

ISI total scores were positively related to age (*r* = 0.206), FBG (*r* = 0.169), positive (*r* = 0.234), negative (*r* = 0.181), depressive factor scores (*r* = 0.261), PANSS total scores (*r* = 0.167), and were negatively correlated with physical activity levels (*r* = –0.247), significantly. After controlling for other variables related to ISI total scores, ISI total scores were still negatively correlated with physical activity levels (*r* = –0.168, *P* = 0.027) (see Table 2).

**TABLE 2:** Correlation analysis of clinical and biological indicators with ISI total scores and HAMD total scores.

Variables	ISI total scores		HAMD total scores	
	***r***	***P***	***r***	***P***
Age (years)	0.206	**0**.**006**	0.145	0.052
Gender	0.084	0.262	0.050	0.504
Education (years)	-0.076	0.311	-0.147	0.050
Illness duration (years)	0.062	0.413	0.063	0.402
Number of admissions	-0.082	0.273	-0.177	**0**.**018**
BMI	0.060	0.427	-0.045	0.552
Monotherapy/ Polypharmacy	0.111	0.138	0.118	0.117
FBG (*mmol/L*)	0.169	**0**.**024**	0.048	0.527
TC(mmol/L)	0.098	0.191	0.013	0.862
TG (mmol/L)	-0.011	0.884	-0.049	0.514
HDL-C (mmol/L)	0.129	0.087	0.000	0.996
LDL-C (mmol/L)	0.109	0.149	-0.020	0.791
Physical activity levels	-0.247	**0**.**001**	-0.312	**<0**.**001**
Positive factor scores	0.234	**0**.**002**	0.203	0.006
Negative factor scores	0.181	**0**.**016**	0.316	**<0**.**001**
Cognitive factor scores	0.077	0.305	0.251	**0**.**001**
Excited factor scores	0.085	0.259	0.116	0.122
Depressive factor scores	0.261	**<0**.**001**	0.506	**<0**.**001**
PANSS total scores	0.167	**0**.**026**	0.314	**<0**.**001**
Chlorpromazine equivalent (mg/d)	0.117	0.118	0.112	0.134
Hypnotics prescription	0.000	0.996	0.058	0.440
Antidepressants prescription	-0.014	0.852	0.025	0.741

Bold value means *P* < 0.05.

HAMD total scores were positively correlated with negative (*r* = 0.316), cognitive (*r* = 0.251), depressive (*r* = 0.506), PANSS total scores (*r* = 0.314), and were negatively correlated with number of admissions (*r* = –0.177) and physical activity levels (*r* = –0.312), significantly. After controlling for other variables related to HAMD total scores, HAMD total scores were still negatively correlated with physical activity levels (*r* = –0.188, *P* = 0.013) (see Table 2).

### Influencing factors associated with insomnia symptoms in CS patients

The results showed that older age (*OR* = 1.07, *95% CI*: 1.02–1.14, *P* = 0.013),vigorous physical activity (compared to physical inactivity) (*OR* = 0.22, *95% CI*: 0.06–0.88, *P* = 0.032), and higher depressive factor scores (*OR* = 1.22, *95% CI*: 1.02–1.46, *P* = 0.030) were independently correlated with insomnia symptoms in CS patients (see Table 3).

**TABLE 3:** Demographic and clinical variables independently associated with insomnia symptoms by binary logistic regression analysis.

Variables	*B*	*P*	*OR*	*95% CI*
Age	0.072	**0**.**013**	1.07	1.02–1.14
FBG (mmol/L)	0.095	0.535	1.10	0.82–1.48
Physical activity levels (ref. physical inactivity)		0.098		
Moderate physical activity	-0.264	0.547	0.77	0.33–1.81
Vigorous physical activity	-1.517	**0**.**032**	0.22	0.06–0.88
Positive factor scores	0.071	0.297	1.07	0.94–1.23
Negative factor scores	0.038	0.421	1.04	0.95–1.14
Depressive factor scores	0.200	**0**.**030**	1.22	1.02–1.46
PANSS total scores	-0.025	0.241	0.98	0.94–1.02

Bold value means *P* < 0.05.

### Influencing factors associated with depressive symptoms in CS patients

The results found that vigorous physical activity (compared to physical inactivity) (*OR* = 0.19, *95% CI*: 0.07–0.48, *P* = 0.001), higher negative factor scores (*OR* = 1.10, *95% CI*: 1.02–1.19, *P* = 0.019) and depressive factor scores (*OR* = 1.52, *95% CI*: 1.21–1.91, *P* < 0.001) were related to depressive symptoms in CS patients (see Table 4).

**TABLE 4:** Demographic and clinical variables independently associated with depressive symptoms by binary logistic regression analysis.

Variables	*B*	*P*	*OR*	*95% CI*
Number of admissions	-0.038	0.216	0.96	0.91–1.02
Physical activity levels(ref. physical inactivity)		0.002		
Moderate physical activity	-0.873	0.057	0.42	0.17–1.03
Vigorous physical activity	-1.679	**0**.**001**	0.19	0.07–0.48
Negative factor scores	0.096	**0**.**019**	1.10	1.02–1.19
Cognitive factor scores	0.002	0.983	1.00	0.82–1.22
Depressive factor scores	0.418	**<0**.**001**	1.52	1.21–1.91
PANSS	-0.017	0.344	0.98	0.95–1.02

Bold value means *P* < 0.05.

## Discussion

This study revealed that (1) insomnia and depressive symptoms in middle-aged and elderly CS inpatients were 22.9 and 71.5%. Patients with insomnia symptoms tend to be older, have lower levels of physical activity, higher rates of antipsychotic medication combination, higher FBG levels, and higher psychopathological symptoms such as positive, depressive factor scores, PANSS, ISI, and HAMD total scores. Patients with depressive symptoms tended to have fewer hospitalizations, lower levels of physical activity, higher positive, negative, cognitive, and depressive factor scores, PANSS, ISI, and HAMD total scores. (2) After controlling for potential confounders, the ISI total scores and HAMD total scores were still negatively correlated with physical activity levels. (3) Logistic regression analysis revealed that older age, higher depressive factor scores, and lower physical activity level were influential factors of insomnia symptoms in CS patients. In addition, vigorous physical activity (compared with physical inactivity) and higher negative and depressive factor scores were independently related to depressive symptoms in CS patients.

Our research noted that 22.9% of middle-aged and elderly CS patients had insomnia symptoms, which was more consistent with previous studies (7, 8). In addition, older age, higher depressive factor scores, and lower physical activity were related factors of insomnia symptoms. Previous studies pointed out that compared to the general population, schizophrenic patients had less sleep, and the prevalence of sleep-related problems was significantly higher. In contrast, patients with sleep problems had significantly lower life satisfaction and happiness (34). In a study of outpatients with schizophrenia, a high prevalence of sleep disorders was strongly associated with older age, physical inactivity, and severe psychopathological symptoms (9).

Historical literature suggested that physical inactivity was strongly correlated with poorer psychosomatic health in patients with schizophrenia (18). In contrast, regular physical activity significantly improved patients’ insomnia symptoms and their subjective sleep quality to a consistent degree (14, 35). A randomized controlled study in Germany noted that patients with regular physical activity had significantly better sleep quality than psychiatric patients with physical inactivity (36). Second, patients with adequate physical activity had lower rates of insomnia symptoms than those with physical inactivity (37, 38). In addition, schizophrenic patients who underwent weekly physical activity had less overall symptom severity and a higher quality of life than conventional treatment modalities without physical activity (39). A systematic review also indicated that higher levels of physical activity predicted a moderate increase in patients’ motor activity (40) and that increased motor activity improved patients’ insomnia symptoms and cognitive-related impairments (41) and was strongly associated with fewer negative symptoms and depressive symptoms (42). Furthermore, analysis of studies has shown that the beneficial effects of physical activity on sleep quality in psychiatric patients were attributed to its improvement of the overall health status of patients (43). The adjunctive use of physical activity interventions in schizophrenic populations has clear efficacy and no associated side effects (44).

In this study, we found that 71.5% of middle-aged and elderly CS patients had depressive symptoms, and depressive symptoms were independently correlated with severe psychopathological symptoms such as (negative, cognitive, depressive factor scores, PANSS total scores), fewer hospital admissions, and lower physical activity levels. In addition, vigorous physical activity (compared to physical inactivity) and higher negative or depressive factor scores were related to depressive symptoms in CS patients. A study noted that the prevalence of depressive symptoms as a usual comorbidity of schizophrenia in the elderly ranged from 44 to 75% (45). Two studies of older Chinese patients with schizophrenia noted the incidence of depressive symptoms of 48.5% (patients aged: above 65 years old) (46) and 32.8% (patients aged: above 60 years old) (47), both using the Geriatric Depression Scale (GDS) to evaluate depressive symptoms. In addition, a Tunisian study assessed by Depression Anxiety and Stress scales (DASS_–21_) noted that older patients with schizophrenia (age: 66.9 ± 3.8 years old) had a prevalence of comorbid moderate-to-severe depressive symptoms of only about 25% (48). A French study that used the Center of Epidemiologic Studies Depression (CESD) scale to assess depressive symptoms in patients found that 78.1% presented with subsyndromes or syndromes depressive symptoms which were positively related to psychotic symptoms and were not associated with psychotropic medication use, or with the use of antidepressants (49). The results of our research were consistent with those of previous studies by using the HAMD_–17_ scale to assess depressive symptoms. Previous studies have shown that weekly physical activity levels were negatively related to depressive symptoms in schizophrenic patients (50). Furthermore, evidence indicated that regular physical activity significantly reduced depressive symptoms in older adults (51), mainly because patients who engaged in regular physical activity had significantly reduced psychiatric symptoms and fewer physical complaints (52). Physical activity improved the prognosis of quality of schizophrenic patients, resulting in fewer accompanying depressive symptoms (53). According to a national survey in China, compared to population on physical activity in the last 1 year, people who sometimes or often participated in physical activity had significantly lower symptoms of depression and anxiety (24). Moreover, a systematic review indicated that compared to adults engaged in lower level of physical activity, MVPA was associated with better mental health and higher quality of life (25). The previous solid conclusions suggested that physical activity could be routinely incorporated into the regular treatment and daily care of patients with mental disorders because of its clear benefits (54).

Although current evidence pointed to a beneficial role of physical activity on psychopathological symptoms, insomnia, or depressive symptoms in CS patients, schizophrenic patients spend significantly less time physically active than the general population (10, 55), with only about one-fifth of patients meeting the minimum required level of physical activity (56). In contrast, physical inactivity associated with sedentary behavior in schizophrenic patients was closely related to a higher prevalence of depressive symptoms (57). Furthermore, due to their negative and depressive symptoms and lack of internal drive, patients with severe mental disorders obtained a lower level of physical activity (58). The relationship between depressive symptoms and physical activity levels is a two-way process (59). Moreover, the high rate of sleep disturbance in schizophrenic patients, resulting in reduced energy and deficits, was also associated with physical inactivity (60).

The Study on Global Ageing and Adult Health (SAGE) demonstrated that meeting physical activity guidelines (≥ 150 min of MVPA/week) was significantly associated with more happiness (61). A systematic review of 31 studies found that reducing sedentary behavior and increasing MVPA were significantly associated with better mental health and quality of life (25). Our study indicated that the prevalence of insomnia and depressive symptoms was high in CS patients. Furthermore, higher level of physical activity correlated to fewer insomnia and depressive symptoms. In clinical treatment, in addition to conventional pharmacotherapy or psychotherapy, physical activity can also be used to relieve the emotional symptoms of patients with mental disorders.

This study has the following limitations. First of all, this study was a cross-sectional study, which only explored the correlation between physical activity levels and insomnia and depressive symptoms of the enrolled patients, and could not be determined the causality. Second, the enrolled subjects were middle-aged and older adults, and the prevalence of depressive symptoms was slightly higher than that of the elderly reported in previous studies, which might be due to the differences in the age of the subjects or the assessment tools, which was also the innovation of this study. Third, although combined with electronic medical records as an objective reference, the physical activity level of the subjects in this study was assessed using subjective questions. Using objective methods like accelerometers will give stronger corroborating evidence than subjective description of the physical activity by patients. Moreover, the small sample size included in this study could lead to the bias of the study results, and further large-sample controlled studies are needed.

## Conclusion

This study investigated the association between physical activity levels, insomnia, and depressive symptoms in middle-aged and elderly patients with CS. The results pointed out that lower physical activity levels were influential factors in comorbid insomnia and depressive symptoms in CS patients. Due to the advantages of physical activity on psychotic symptoms, we should enhance the use of physical activity as a regular adjunct in clinical treatment or psychiatric care to improve the psychosomatic health and quality of life of patients with psychiatric symptoms.

## Data availability statement

The raw data supporting the conclusions of this article will be made available by the authors, without undue reservation.

## Ethics statement

This study was reviewed and approved by the Biomedical Ethics Committee of Chaohu Hospital of Anhui Medical University (Grant no. 201805-kyxm-03). The patients/participants provided their written informed consent to participate in this study. Written informed consent was obtained from the individual(s) for the publication of any potentially identifiable images or data included in this article.

## Author contributions

ZL, YZ, LS, JW, and HL collected and statistically analyzed the data and wrote the first draft. All authors revised and approved the manuscript.
